# TKI Maintenance After Stem-Cell Transplantation for *FLT3*-ITD Positive Acute Myeloid Leukemia: A Systematic Review and Meta-Analysis

**DOI:** 10.3389/fimmu.2021.630429

**Published:** 2021-03-12

**Authors:** Nico Gagelmann, Christine Wolschke, Evgeny Klyuchnikov, Maximilian Christopeit, Francis Ayuk, Nicolaus Kröger

**Affiliations:** Department of Stem Cell Transplantation, University Medical Center Hamburg-Eppendorf, Hamburg, Germany

**Keywords:** sorafenib, midostaurin, maintenance, allogeneic stem cell transplantation, FLT3-internal tandem duplication, acute myeloid leukemia, graft-vs.-host disease

## Abstract

This analysis aimed to systematically review and synthesize the existing evidence regarding the outcome of tyrosine kinase inhibitor (TKI) maintenance therapy after allogeneic stem-cell transplantation for patients with *FLT3*-ITD-mutated acute myeloid leukemia (AML). We searched publicly available databases, references lists of relevant reviews, registered trials, and relevant conference proceedings. A total of 7 studies comprising 680 patients were included. Five studies evaluated sorafenib and 2 studies evaluated midostaurin, compared with control. The incidence of relapse was significantly reduced after TKI therapy, showing an overall pooled risk ratio (RR) of 0.35 (95% confidence interval [CI], 0.23-0.51; *P* < 0.001), with a marked 65% reduced risk for relapse. The overall pooled RR for relapse-free survival and overall survival showed significantly improved outcome after TKI maintenance therapy, being 0.48 (95% CI, 0.37–0.61; *P* < 0.001) and 0.48 (95% CI, 0.36–0.64; *P* < 0.001). The risk for relapse or death from any cause was reduced by 52% using TKI. No difference in outcome was seen for non-relapse mortality, and the risk for chronic or acute graft-vs. -host disease appeared to be increased, at least for sorafenib. In conclusion, post-transplant maintenance therapy with TKI was associated with significantly improved outcome in relapse and survival in patients with *FLT3*-ITD positive AML.

## Introduction

Acute myeloid leukemia (AML) is a heterogeneous hematologic malignancy derived from hematopoietic stem cells with a series of abnormalities on the level of cytogenetics, genetics, and epigenetics (1, 2). Prognosis of this disease varies widely according to mutation profile, patient age, and comorbidities (2, 3). The duplication in Fms-like tyrosine kinase 3-internal tandem (*FLT3*-ITD) occurs in about 25% of adult AML patients (4–7). Patients harboring *FLT3*-ITD, particularly those with a high allelic ratio, show increased relapse rates and inferior survival, despite undergoing allogeneic stem-cell transplantation (6, 8).

In the front-line setting of *FLT3*-mutated AML, combining conventional chemotherapy with a multi-targeted tyrosine kinase inhibitor (TKI), namely midostaurin, resulted in improved overall survival (9). Another multi-targeted TKI, sorafenib, has been approved for solid tumors such as hepatocellular and renal cell cancer (10, 11), but it has also shown efficacy in terms of prolonged progression-free survival in younger AML patients in combination with upfront chemotherapy (12), but not in the elderly population (13). In the relapsed/refractory setting, patients with *FLT3*-ITD-positive AML receiving TKI monotherapy showed promising outcomes (14–16), while this approach may remain a palliative strategy which is furthermore limited by emerging TKI resistance (17, 18). In contrast, when patients with *FLT3*-ITD-mutated AML relapsing after allogeneic stem-cell transplantation received sorafenib, the outcome may differ profoundly, as suggested by long-term remissions in selected patients (19, 20).

To reflect the increasing interest within clinical and basic research, we aimed to systematically review the current body of literature and to synthesize the existing evidence regarding the outcome of TKI maintenance therapy after allogeneic stem-cell transplantation for patients with *FLT3*-ITD-mutated AML.

## Methods

The methodology of this systematic review with meta-analysis was undergone in accordance with the Cochrane handbook. Further, dimensions of reporting were assessed with the preferred reporting items for systematic reviews and meta-analyses (PRISMA) guidelines and the meta-analysis of observational studies in epidemiology (MOOSE) checklist and adhered accordingly (21, 22). The research question was defined using the PICOS framework: population, FLT3-ITD mutated AML; intervention, stem-cell transplantation with TKI maintenance; comparator, placebo, or no maintenance; outcome, survival and relapse; study design, retrospective and prospective comparative studies.

### Search Strategy

Medline and the Cochrane Library were searched (until August 11, 2020, respectively). Additionally, meeting abstracts archived between 2017 and 2020 from hematology/oncology meetings were screened. Review of clinicaltrials.gov was performed until August 11, 2020. The search strategy consisted of keywords specific to each database and considered all trial designs of human subjects and was not restricted by language. Search terms included all subject headings and associated keywords for “sorafenib or midostaurin or gilteritinib” and “leukemia or leukemia.” Reference lists of relevant reports were reviewed in addition.

### Study Selection, Data Extraction, and End Points

Two reviewers (NG and NK) independently screened titles, abstracts, and the full text of relevant articles. Disagreements were resolved by consensus. Studies were included if they fulfilled the following criteria: adult patients with *FLT3*-ITD AML; prospective or retrospective studies reporting on patients receiving TKI therapy after stem-cell transplantation; evaluating a comparison with a control; reporting at least on relapse-free survival and/or cumulative incidence of relapse.

The following information was extracted from the included studies: the name of the first author, year of publication, study design, TKI treatment, control, number of participants, conditioning intensity for stem-cell transplantation, frequency of high-risk cytogenetics within the studied population, length of follow-up, and primary, and secondary outcomes. Primary end points for data synthesis were relapse-free survival and cumulative incidence of relapse. Secondary end points were overall survival, non-relapse mortality, chronic and acute graft-vs. -host disease (GVHD). Relapse-free survival was defined as time from randomization to first event of either AML relapse or death from any cause in prospective studies or as defined in retrospective studies. Definition of relapse was used in accordance with the included studies.

### Risk of Bias and Quality Assessment

Risk of bias for prospective trials was addressed in accordance with tools developed by the Cochrane Collaboration, and the risk of bias for retrospective comparisons was assessed using the ROBINS-I tool (23). The certainty of the evidence for each outcome was assessed using the grading of recommendations assessment, development, and evaluation (GRADE) approach (24), including considerations of risk of bias, inconsistency, indirectness, imprecision, and publication bias. Retrospective studies were judged a priori as having serious risk of bias, in accordance with the GRADE approach. The resulting overall certainty of the evidence was assessed as high, moderate, low, or very low. All end points within the quality assessment were considered as being of critical importance.

### Data Synthesis and Analysis

Risk ratios (RRs) and 95% confidence intervals (CIs) were calculated for primary and secondary end points by pooling the results from studies using the Mantel-Haenszel method and the random-effects model. Heterogeneity was assessed using *I*^2^ and was categorized from moderate to high (25). Prespecified subgroups were different TKIs (midostaurin and sorafenib). All values with *P* < 0.05 were considered statistically significant. Means were calculated for the end point of safety. Analyses were performed using R statistical software version 3.6.1 using the meta and metafor packages (R Core Team. R: A language and environment for statistical computing. R Foundation for Statistical Computing, Vienna, Austria. https://www.R-project.org/)(26).

## Results

### Search Results

**Figure 1:**
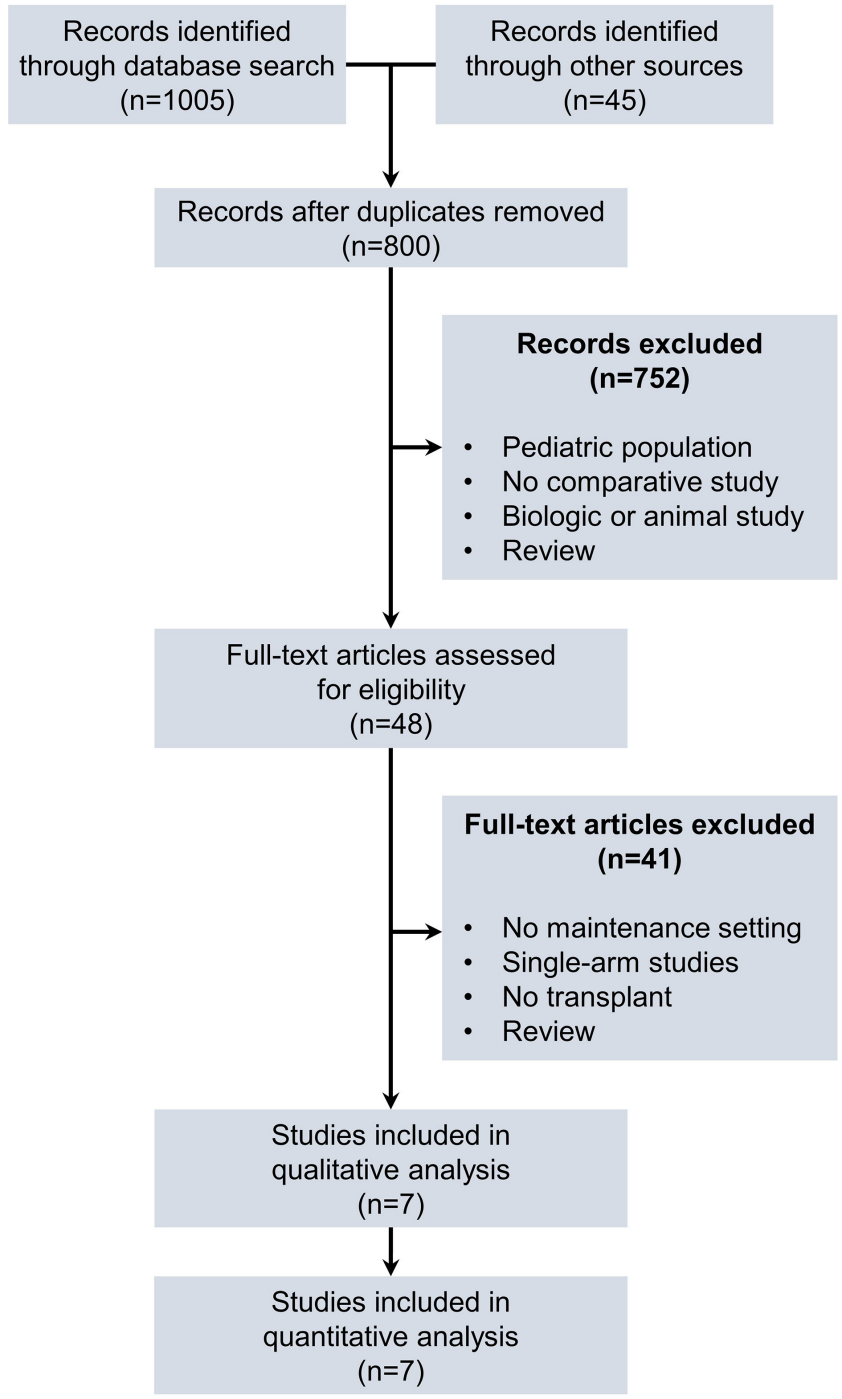
Study selection process.

A total of 1050 citations were identified from the electronic database search and from other sources including meeting abstracts. After duplicates were removed, 800 unique citations remained. Based on title and abstract screening, 752 citations were excluded. Forty-one citations were excluded on the basis of screening full-text articles. Reasons for exclusion were: studies with no maintenance setting; lack of direct comparison results; no patients undergoing allogeneic stem-cell transplantation; and review articles. Seven studies (27–33) were included in qualitative and quantitative analyses (Figure 1).

### Study Characteristics and Risk of Bias

A total of 680 patients were included in the 7 studies. Three studies (28, 31, 32) were prospective randomized studies and 1 study (29) was a prospective study that compared TKI intervention with historical controls using propensity score matching. One prospective study was an abstract, and fully published data were not accessible during finalization of the present manuscript (32). The remaining 3 studies (27, 30, 33) were of retrospective design. Five studies evaluated the efficacy of sorafenib comprising 504 patients while the remaining 2 studies evaluated the TKI midostaurin and comprised 176 patients. Median age in the TKI group ranged from 24 to 55 years and frequency of patients having complete remission at time of transplantation in the TKI group ranged from 61 to 100%. Four studies only used myeloablative conditioning transplantation. Median time of follow-up ranged from 18 to 59 months. The remaining characteristics are summarized in Table 1.

**Table 1:** Study characteristics.

**Study**	**Design**	**N**	**Age in TKI group (range)**	**TKI**	**Comparator**	**Myeloablative conditioning**	**CR at transplant^b^**	**High-risk cytogenetics**	**Length of follow-up**
Burchert et al. (31)	Randomized phase 2	83	54 (23–74)	Sorafenib	Placebo	TKI: 42%, Placebo: 47%	TKI: 63%, placebo: 48%	TKI: 2%, Placebo: 8%	42 months
Brunner et al. (30)	Retrospective	81	55 (20–74)	Sorafenib	No TKI	TKI: 54%, No: 49%	100% (CR1)	8%	27 months
Schlenk et al. (29)	Prospective phase 2, propensity score matching with historical controls	116^a^	54 (18–70)	Midostaurin	Historical control	NR	TKI: 61%, control: 43%	NR	24 months
Xuan et al. (28)	Randomized phase 3	202	35 (26–42)	Sorafenib	No TKI	100%	TKI: 73%, no: 77%	TKI: 7%,no: 5%	21 months
Xuan et al. (27)	Retrospective	82	37 (15–55)	Sorafenib	No TKI	100%	77%	TKI: 6%, no: 1%	59 months
Maziarz et al. (32)	Randomized phase 2	60	18–70^c^	Midostaurin	No TKI	100%	NR	NR	18 months
Shi et al. (33)	Retrospective	56	24 (14–62)	Sorafenib	No TKI	100%	100%	17%	24 months

*N, number; TKI, tyrosine kinase inhibitor; HSCT, hematopoietic stem-cell transplantation; MAC, myeloablative conditioning; RIC, reduced intensity conditioning; CR, complete remission; NR, not reported*.

a *The original number of patients in the study was 284, here we report on the subgroup analyses of patients that actually underwent midostaurin maintenance after stem-cell transplantation or not*.

b *As reported in the patient characteristics of the trials*.

c *Inclusion criteria, age distribution not given*.

The duration of maintenance treatment differed between the studies. Maintenance was administered for 24 months or until occurrence of relapse, or limiting toxicity in Burchert et al. (31) In both studies from Xuan et al. (27, 28) TKI was given until day 180 after transplantation or until intolerable adverse events occurred. Maziarz et al. (32) applied TKI for twelve 4-week cycles. Patients in the study from Shi et al. (33) received TKI maintenance at a median of 238 days (range, 21–385 days). In Brunner et al. (30) TKI therapy was planned for 12–24 months, leaving continuation or early withdrawal to the discretion of the treating physician. Schlenk et al. (29) gave TKI therapy for 365 days.

Low risk of bias was assessed in 2 prospective randomized studies (28, 31), 4 studies showed moderate risk of bias (16, 27, 30, 33), and 2 studies conferred high risk of bias (29, 32). Overall, the risk of bias of the included studies according to each end point was judged to be serious. Publication bias could not be assessed due to the number of < 10 studies included in the analysis, which is in accordance with the Cochrane handbook recommendations. Supplementary Tables 1, 2 depict the summary of the risk of bias profile for each dimension within each study and Supplementary Table 3 summarizes the quality of evidence for each end point.

### Relapse-Free Survival and Incidence of Relapse

**Figure 2:**
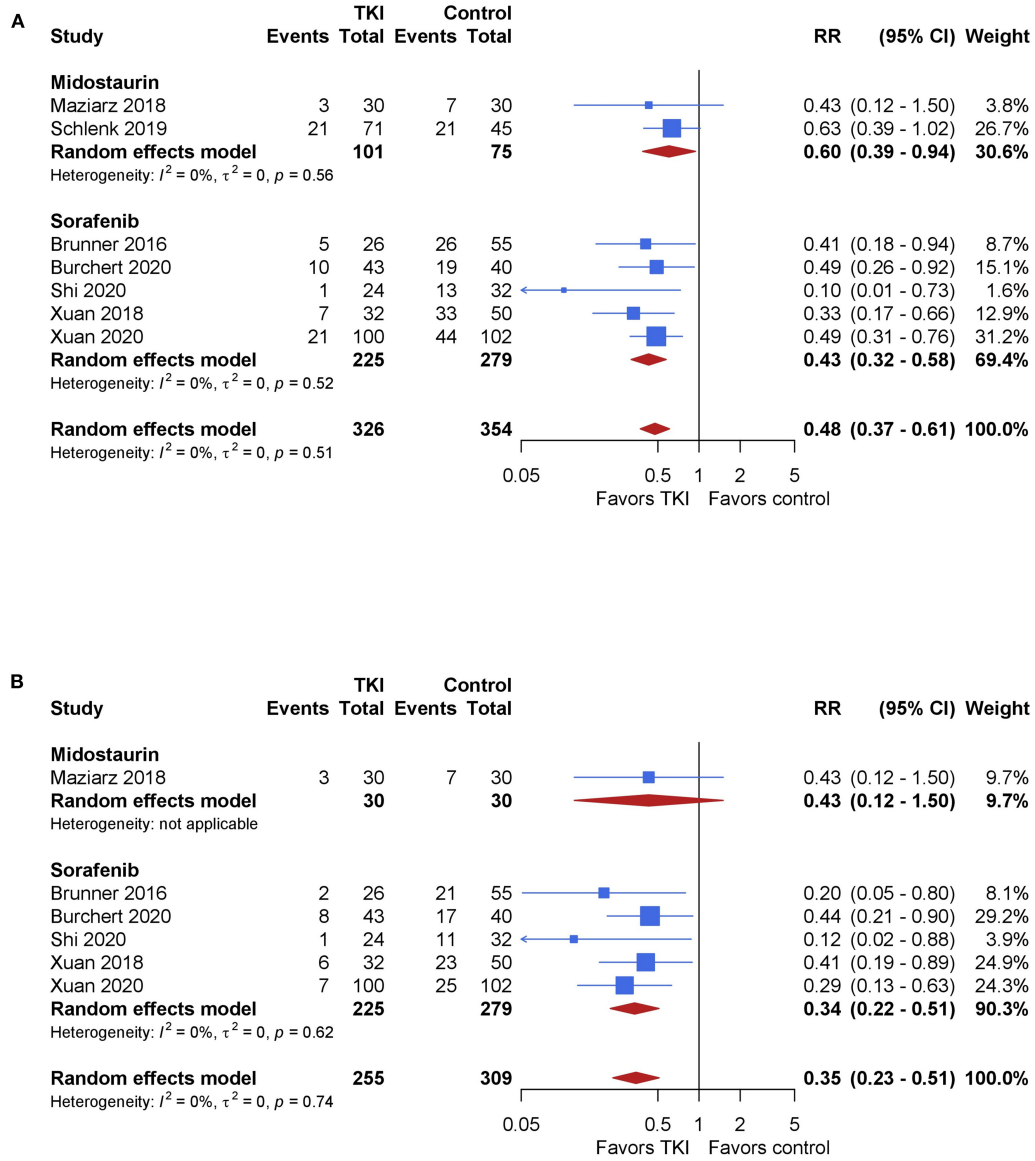
The impact of TKI therapy on primary end points of relapse-free survival and cumulative incidence of relapse. Relapse-free survival **(A)** was assessed in all 7 studies at 18–59 months follow-up. The overall pooled RR showed significantly better relapse-free survival after TKI therapy, being 0.48 (95% CI, 0.37–0.61; *P* < 0.001) with no relevant heterogeneity (*I*^2^ = 0%). Subgroup analyses showed no significant difference in outcome between midostaurin and sorafenib (*P* = 0.21). Incidence of relapse **(B)** was assessed in six studies. The overall pooled RR showed significantly reduced incidence of relapse, being 0.35 (95% CI, 0.23–0.51; *P* < 0.001) in favor of the TKI therapy with no relevant heterogeneity (*I*^2^ = 0%). Subgroup analyses showed no significant difference in outcome between midostaurin and sorafenib (*P* = 0.72).

The primary end point of relapse-free survival was assessed in all 7 studies at 18–59 months follow-up. The overall pooled RR showed significantly better relapse-free survival after TKI therapy, being 0.48 (95% CI, 0.37–0.61; *P* < 0.001) with no relevant heterogeneity (*I*^2^ = 0%, Figure 2A). The quality of the evidence was high. Subgroup analyses showed no significant difference in outcome between midostaurin and sorafenib (*P* = 0.21). However, the pooled RR for midostaurin was 0.60 (95% CI, 0.39–0.94; *I*^2^ = 0%) while a larger effect was seen for sorafenib, being 0.43 (95% CI, 0.32–0.58; *I*^2^=0%), compared with control.

**Figure 3:**
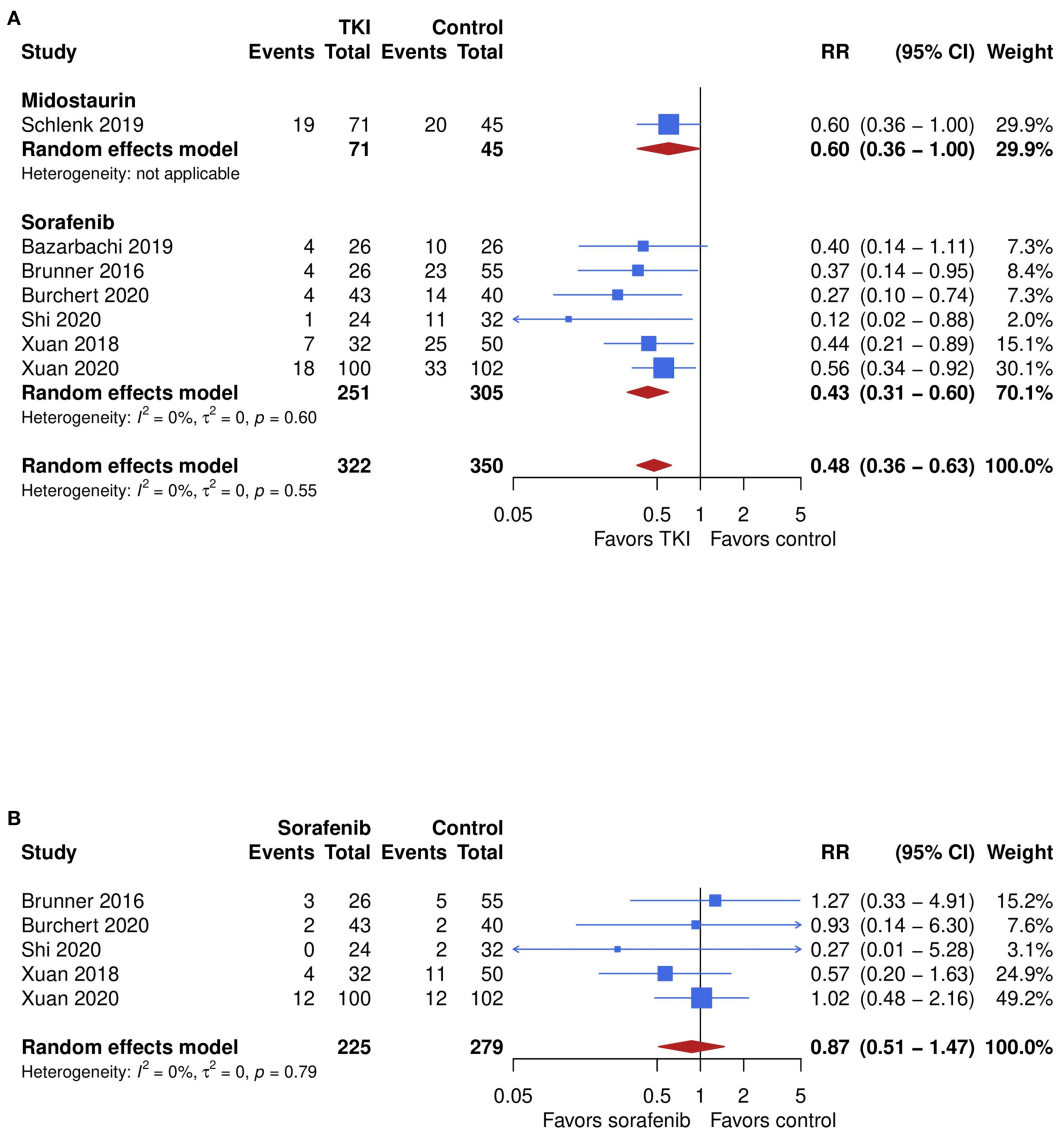
The impact of TKI therapy on secondary end points of overall survival and non-relapse mortality. Significantly improved outcome for TKI therapy was also seen in overall survival **(A)**, which was assessed in 6 studies. The overall pooled RR was 0.48 (95% CI, 0.36–0.64; *P* < 0.001) in favor of the TKI therapy with no relevant heterogeneity (*I*^2^ = 0%). Subgroup analyses showed no significant difference in outcome between midostaurin and sorafenib (*P* = 0.30). Non-relapse mortality **(B)** was assessed in 5 studies, which evaluated the efficacy of sorafenib. No significant difference between sorafenib and the control was seen, showing an overall pooled RR of 0.87 (95% CI, 0.51–1.47; *P* = 0.60) with no relevant heterogeneity (*I*^2^ = 0%).

Incidence of relapse was assessed in six studies. The overall pooled RR showed significantly reduced incidence of relapse, being 0.35 (95% CI, 0.23–0.51; *P* < 0.001) in favor of the TKI therapy with no relevant heterogeneity (*I*^2^ = 0%, Figure 3A). The quality of the evidence was high. Subgroup analyses showed no significant difference in outcome between midostaurin and sorafenib (*P* = 0.72). One study evaluated midostaurin, with a pooled RR of 0.43 (95% CI, 0.12–1.50). Sorafenib showed significantly reduced incidence of relapse showing a RR 0.34 (95% CI, 0.22–0.51; *I*^2^ = 0%), compared with control.

### Overall Survival and Non-relapse Mortality

Significantly improved outcome for TKI therapy was also seen in overall survival, which was assessed in 6 studies. The overall pooled RR was 0.48 (95% CI, 0.36-0.64; *P* < 0.001) in favor of the TKI therapy with no relevant heterogeneity (*I*^2^ = 0%, Figure 2B). The quality of the evidence was high. Subgroup analyses showed no significant difference in outcome between midostaurin and sorafenib (*P* = 0.30). The pooled RR for midostaurin, which was evaluated only in 1 study, was 0.60 (95% CI, 0.36–1.00). A larger effect was seen for sorafenib after synthesis of the remaining 7 studies, with a RR 0.48 (95% CI, 0.36–0.64; *I*^2^ = 0%), compared with control.

Non-relapse mortality was assessed in 5 studies, which evaluated the efficacy of sorafenib. No significant difference between sorafenib and the control was seen, showing an overall pooled RR of 0.87 (95% CI, 0.51–1.47; *P* = 0.60) with no relevant heterogeneity (*I*^2^ = 0%, Figure 3B). The quality of the evidence was low.

### Graft-vs.-Host Disease and Safety

**Figure 4:**
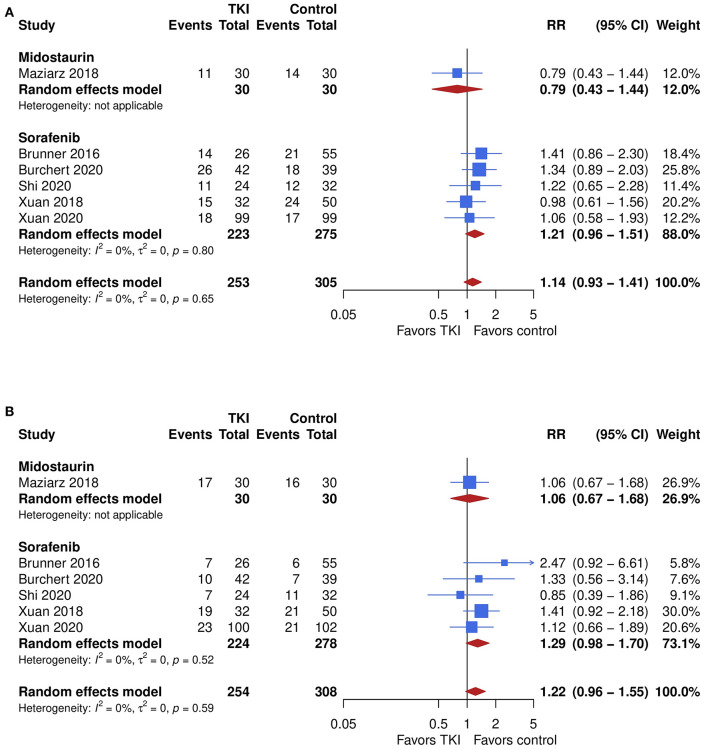
The impact of TKI therapy on secondary end points of acute and chronic GVHD. Chronic GVHD **(A)** was assessed in six studies. No significant difference in the incidence was seen, with a trend toward higher incidence after TKI therapy showing an overall pooled RR of 1.14 (95% CI, 0.93–1.41; *P* = 0.21) with no relevant heterogeneity (*I*^2^ = 0%). Subgroup analyses showed no significant difference in outcome between midostaurin and sorafenib (*P* = 0.19). However, the pooled RR for midostaurin was 0.79 (95% CI, 0.43–1.44) while results for sorafenib suggested higher risk for chronic GVHD showing a RR of 0.43 (95% CI, 0.32–0.57; *I*^2^ = 0%), compared with control. Similar results were yielded for acute GVHD **(B)**, which was assessed in six studies. The overall pooled RR was 1.22 (95% CI, 0.96–1.55; *P* = 0.10) with no relevant heterogeneity (*I*^2^ = 0%). No difference was seen between the TKIs (*P* = 0.48). One study which evaluated midostaurin showed a RR of 1.06 (95% CI, 0.67–1.68), while risk for acute GVHD appeared to be increased after sorafenib therapy showing a RR of 1.29 (95% CI, 0.98–1.70; *I*^2^ = 0%), when compared with control.

Chronic GVHD was assessed in 6 studies. No significant difference in the incidence was seen, with a trend toward higher incidence after TKI therapy showing an overall pooled RR of 1.14 (95% CI, 0.93–1.41; *P* = 0.21) with no relevant heterogeneity (*I*^2^ = 0%, Figure 4A). The quality of the evidence was low. Subgroup analyses showed no significant difference in outcome between midostaurin and sorafenib (*P* = 0.19). However, the pooled RR for midostaurin was 0.79 (95% CI, 0.43–1.44) while results for sorafenib suggested higher risk for chronic GVHD showing a RR of 0.43 (95% CI, 0.32–0.57; *I*^2^ = 0%), compared with control.

Similar results were yielded for acute GVHD, which was assessed in six studies. The overall pooled RR was 1.22 (95% CI, 0.96–1.55; *P* = 0.10) with no relevant heterogeneity (*I*^2^ = 0%, Figure 4B). The quality of the evidence was high. No difference was seen between the TKIs (*P* = 0.48). One study which evaluated midostaurin showed a RR of 1.06 (95% CI, 0.67–1.68), while risk for acute GVHD appeared to be increased after sorafenib therapy showing a RR of 1.29 (95% CI, 0.98–1.70; *I*^2^ = 0%), when compared with control.

The safety profile could be assessed in the two randomized controlled trials on sorafenib (28, 31), for which means were calculated (Table 2). Frequency of adverse events were mostly comparable while skin toxicity was seen more frequently in the sorafenib group (19.5%) in comparison with the control group (6.3%), and hematologic toxicities such as neutropenia and thrombocytopenia, albeit in low absolute numbers, were more frequently observed in the sorafenib group (8.7 and 8.9%) compared with the control group (4.8 and 4.3%).

**Table 2:** Safety of sorafenib in 2 randomized controlled trials.

	**Sorafenib**	**Control**
	**(*n* = 143)**	**(*n* = 142)**
Neutropenia	8.7%	4.8%
Thrombocytopenia	8.9%	4.3%
Skin toxicity	19.5%	6.3%
Infections	29.6%	28.0%
Gastrointestinal toxicity	25.2%	21.7%
Cardiac and renal insufficiency	11.8%	7.8%

## Discussion

Patients with *FLT3*-ITD mutated AML undergoing allogeneic stem-cell transplantation have a high risk of relapse (34). Because oncogenic addiction is caused by *FLT3*-ITD (35), it was reasonable to hypothesize that it could be a potential therapeutic target in *FLT3*-ITD mutated patients (36). While evidence accumulated that the multi-targeted TKI midostaurin can improve outcome in the front-line setting (9), whether specifically targeting *FLT3*-ITD using TKI therapy after allogeneic stem-cell transplantation can improve outcome was long unknown (6, 37, 38).

This first evidence synthesis for TKI therapy after allogeneic stem-cell transplantation in *FLT3*-ITD mutated AML found TKI therapy using midostaurin or sorafenib in comparison with control was significantly associated with better outcome in relapse and relapse-free survival. The risk for relapse was reduced by marked 65% and the risk for relapse or death from any cause was reduced by 53% using TKI. Furthermore, overall survival was significantly improved after TKIs with a risk reduction for death from any cause by 52%. No significant difference for non-relapse mortality was noted, which was only assessed in studies on sorafenib. The risk for GVHD appeared to be increased for TKI therapy.

Although the results of this analysis did not seem to be influenced by different TKIs, more studies evaluated the role of sorafenib (6). Two studies used midostaurin, of which 1 is a still ongoing phase 2 randomized study and 1 a priori studied the effects of midostaurin throughout the therapeutic course, with a subgroup analysis of post-transplant therapy compared with no post-transplant therapy. Other TKIs, for example, quizartinib and gilteritinib, which inhibit FLT3 more specifically and potently in comparison with midostaurin (39), showed improvement in overall survival in relapsed/refractory patients (18, 40). Gilteritinib is also being investigated for post-transplantation maintenance in AML patients with *FLT3*-ITD in a phase 3 randomized study (NCT02997202). Further research is needed to ascertain the comparative efficacy and safety of different TKIs post-transplantation therapy in *FLT3*-ITD mutated AML.

Given the well-described impact of minimal residual disease (MRD) on the outcomes after allogeneic stem-cell transplantation for AML (41, 42), and with the availability of a commercially available, next-generation sequencing-based MRD test for such patients, demonstration of a benefit of TKI therapy (or control) is critical to develop and incorporate TKIs into risk-based maintenance approaches (43). Both prospective randomized studies on sorafenib showed subgroup results according to the MRD status at time of randomization (28, 31). While the Chinese study group showed significantly reduced incidence of relapse after sorafenib with hazard ratios of 0.28 for patients with undetectable MRD and 0.25 for detectable MRD (28), patients with undetectable MRD appeared to have better relapse-free survival in the German study group, but this comparison was not statistically significant (31). In the German study group, patients with detectable MRD had significantly improved relapse-free survival, while the results need to be interpreted with caution owing to the relatively low numbers of patients in each group. The ongoing BMT CTN 1506 study on gilteritinib includes the critical objective to better understand the impact of MRD on outcomes with post-transplantation TKI maintenance.

Recent basic research findings indicate that the synergism of T-cells and sorafenib may metabolically reprogram AML-reactive T-cells, providing potential to contribute to immune-mediated curative treatment of *FLT3*-ITD mutated AML relapse (44). Furthermore and in general, a graft-vs.-leukemia effect is considered to be associated with the occurrence of GVHD (45). The findings of the present data synthesis suggest that at least sorafenib might increase the incidence of GVHD. Whether other mechanisms are involved in this effect requires further investigation.

In terms of safety, multi-targeted TKIs such as midostaurin and sorafenib are relatively non-specific and exert off-target activities. The prospective study on front-line midostaurin showed no unexpected adverse events (9). Higher grade 3–4 adverse events were seen for anemia (92.7 vs. 87.8%), rash (14.1 vs. 7.6%), and nausea (9.6 vs. 5.6%) in comparison with placebo, with no necessary dose modification for hematologic toxicity. With respect to sorafenib, small-sample studies have shown that the most common adverse events were related to hematological, skin, and gastrointestinal toxicities. In the present analysis, safety of post-transplantation TKI therapy could only be assessed for both prospective studies on sorafenib which showed no unexpected and comparable rates of adverse events when compared with control (Table 2). Only skin toxicity appeared to be slightly increased, but the overlap in skin rashes between an adverse event caused by sorafenib and graft-vs.-host disease of the skin represents a difficulty for the differential diagnosis (27, 46). Furthermore, 60 and 50% of patients in the Chinese and German study needed a dose modification (interruption or reduction) because of adverse events. Dose reductions did not seem to limit sorafenib efficacy but more attention in view of TKI-specific toxicities and dose intensities is needed.

As with any meta-analysis, the present evidence synthesis regarding TKIs after stem-cell transplantation has several limitations. The conditioning intensity for transplantation was not homogenous. Four studies only used myeloablative conditioning transplantation (27, 28, 32, 33). Comparative analyses on the superiority of one conditioning over another are inconclusive and may be interpreted on the subgroup level (42, 47–50), and the evidence on the impact of conditioning on outcome after TKIs is immature (51). Furthermore, the time of initiation of TKI was not homogeneous between studies and this meta-analysis could not account for differences in dosage schemes nor duration of treatment or treatment interruptions. Additionally, the present analysis may not provide any evidence for favoring one TKI over another. Further, RRs had to be calculated at different time of follow-up in the included studies, ranging from 18 to 59 months. This issue can be controlled for only when patient-level data are available. The risk of selection bias in meta-analyses of different donor stem-cell transplantation studies or due to the incorporation of findings from retrospective and prospective studies cannot be completely ruled out (52, 53). One prospective study on midostaurin was not adequately powered to identify a statistical difference between the groups (32), and on prospective study on sorafenib was prematurely terminated owing to slow patient recruitment (31). However, upfront exclusion of certain studies may even increase heterogeneity. And last, associations of allele ratios or TKD mutations cannot be addressed by analyses as presented here and further prospective evaluations are warranted.

In sum, this analysis identified a significant improvement in relapse-free survival, overall survival, and relapse incidence after post-transplant TKI therapy in *FLT3*-ITD mutated AML. These effects are irrespective of the TKI, while there is more consistent evidence for sorafenib so far. Ongoing studies could further help to better dissect patient subgroups that may benefit the most and identify refined relation of FLT3 selectivity vs. immune-stimulatory off-target activities governing TKI therapy after stem-cell transplantation in *FLT3*-ITD mutated AML.

## Data Availability Statement

The original contributions presented in the study are included in the article/Supplementary Material, further inquiries can be directed to the corresponding author/s.

## Author Contributions

NG and NK had full access to all the data in the study, designed the study, retrieved, analyzed and interpreted the data, and wrote the first draft of the manuscript. All authors interpreted the data, wrote the manuscript, and approved the final version.

## Conflict of Interest

NK and CW were co-authors to one included study. The remaining authors declare that the research was conducted in the absence of any commercial or financial relationships that could be construed as a potential conflict of interest.
