# Supplementary material for: TKI Maintenance After Stem-Cell Transplantation for FLT3-ITD Positive Acute Myeloid Leukemia: A Systematic Review and Meta-Analysis
Source: Front Immunol. 2021 Mar 12;12:630429. doi: 10.3389/fimmu.2021.630429 (PMC8006462; doi:10.3389/fimmu.2021.630429)
Supplement: Supplementary file 1 [file Data_Sheet_1.docx]

Supplemental Material

**TKI maintenance after stem-cell transplantation for *FLT3*-ITD positive acute myeloid leukemia**

Nico Gagelmann^1^, Christine Wolschke^1^, Evgeny Klyuchnikov^1^, Maximilian Christopeit^1^, Francis Ayuk^1^, Nicolaus Kröger^1^

^1^ Department of Stem Cell Transplantation, University Medical Center Hamburg-Eppendorf, Hamburg, Germany

Table 1. Risk of bias for prospective studies according to the Cochrane Collaboration risk of bias tool.

Table 2. Risk of bias assessment using the tool for assessing risk of bias in non-randomized studies of interventions (ROBINS-I).

Table 3. Quality of evidence.

**Table 1. Risk of bias for prospective studies.**

| **Study** | **Random sequence generation** | **Allocation concealment** | **Blinding** | **Incomplete outcome data** | **Selective reporting** | **Other bias** |
| --- | --- | --- | --- | --- | --- | --- |
| Burchert 2020 | Low | Low | Low | Low | Low | Low |
| Schlenk 2019 | High | High | High | High | High | High |
| Xuan 2020 | Low | Low | High | Low | Low | Low |
| Maziarz 2018 | Low | Low | High | High | High | High |

**Table 2. Risk of bias assessment using the tool for assessing risk of bias in non-randomized studies of interventions (ROBINS-I).**

| **Study** | **Confounding** | **Selection of participants** | **Classification of participants** | **Deviations from intended interventions** | **Missing data** | **Measurement of outcomes** | **Selection of reported results** | **Overall risk of bias** |
| --- | --- | --- | --- | --- | --- | --- | --- | --- |
| Brunner 2020 | Yes | Yes | Yes | Yes | Yes | Yes | Yes | Low |
| Xuan 2018 | PN | Yes | Yes | Yes | PN | Yes | Yes | Low |
| Shi 2020 | Yes | PN | Yes | Yes | PN | Yes | PN | Moderate |

**Table 3. Quality of evidence.**

| **No. of studies** | **Risk of bias** | **Inconsistency** | **Indirectness** | **Imprecision** | **Publication bias** | **TKI** | **Control** | **Risk ratio (95% CI)** | **Quality** | **Importance** |
| --- | --- | --- | --- | --- | --- | --- | --- | --- | --- | --- |
| **Relapse-free survival** | | | | | | | | | | |
| 7 | Serious ^a^ | Not Serious | Not serious | Not serious | None | 326 | 354 | 0.48  (0.37-0.61) | ⨁⨁⨁⨁  High ^b^ | Critical |
| **Overall survival** | | | | | | | | | | |
| 6 | Serious | Not Serious | Not serious | Not serious | None | 296 | 324 | 0.48  (0.36-0.64) | ⨁⨁⨁⨁  High ^a^ | Critical |
| **Cumulative incidence of relapse** | | | | | | | | | | |
| 6 | Serious | Not Serious | Not serious | Not serious | None | 255 | 309 | 0.35  (0.23-0.51) | ⨁⨁⨁⨁  High ^a^ | Critical |
| **Non-relapse mortality** | | | | | | | | | | |
| 5 | Serious | Not Serious | Not serious | Serious ^c^ | None | 225 | 279 | 0.87  (0.51-1.47) | ⨁⨁◯◯  Low | Critical |
| **Chronic GVHD** | | | | | | | | | | |
| 6 | Serious | Not Serious | Not serious | Serious ^c^ | None | 253 | 305 | 1.14  (0.93-1.41) | ⨁⨁◯◯  Low | Critical |
| **Acute GVHD** | | | | | | | | | | |
| 6 | Serious | Not serious | Not serious | Serious ^c^ | None | 254 | 308 | 1.22  (0.96-1.55) | ⨁⨁◯◯  Low | Critical |
| Abbreviations: TKI, tyrosine kinase inhibitor; CI, confidence interval; GVHD, graft-versus-host disease.  Note: In accordance with GRADE criteria, quality of evidence with respect to each end point was downgraded a priori starting at moderate quality of evidence, because most studies had a retrospective design.  ^a^ Serious risk of bias: only 1 prospective study showed low risk of bias in all dimension; 1 prospective study is of high risk of bias, given only subgroup analysis could be evaluated; 3 studies included in the analysis are of retrospective design, with probable missing data.  ^b^ We upgraded due to strong effect.  ^c^ CI crosses decision-making threshold. | | | | | | | | | | |
